# 2,3,5,4′-Tetrahydroxystilbene-2-O-*β*-D-glucoside Promotes Expression of the Longevity Gene Klotho

**DOI:** 10.1155/2016/3128235

**Published:** 2016-11-03

**Authors:** Shuang Ling, Ju Duan, Rongzhen Ni, Jin-Wen Xu

**Affiliations:** Institute of Interdisciplinary Research Complex, Shanghai University of Traditional Chinese Medicine, Shanghai 201203, China

## Abstract

The longevity gene klotho has numerous physiological functions, such as regulating calcium and phosphorus levels, delaying senescence, improving cognition, reducing oxidative stress, and protecting vascular endothelial cells. This study tested whether 2,3,5,4′-Tetrahydroxystilbene-2-O-*β*-D-glucoside (THSG), a small molecule with antiaging effects, regulates the expression and physiological effects of klotho. Our results showed that THSG dose-dependently increased the luciferase reporter activity of the klotho gene, reversed the decrease in mRNA and protein expression of klotho which was induced by angiotensin II in NRK-52E renal tubular epithelial cells, and increased klotho mRNA expression in the cerebral cortex, hippocampus, testis, and kidney medulla of spontaneously hypertensive rats. THSG also reduced the number of senescent cells induced by angiotensin II and improved the antioxidant capacity and enhanced the bone strength in vivo. Based on klotho's role in promoting cognition, regulating bone metabolism, and improving renal function, the effect of THSG on klotho expression will be beneficial to the functional improvement or enhancement of the expressed organs or tissues.

## 1. Introduction

Klotho is a longevity gene that was discovered in 1997 which is expressed in various organs and cells, such as renal tubular cells and the brain choroid [1]. The *α*-klotho protein can be cleaved, secreted, and released into circulation, presenting a hormone-like effect [2]. Klotho as a serum secretory protein is closely related to age. Its serum level is low in the first decade; it then increases and reaches its peak in the 20–40 age groups and thereafter gradually decreases with age [3]. Klotho has many physiological functions, such as regulating calcium and phosphorus levels in vivo, delaying senescence, improving cognition, reducing oxidative stress, and protecting vascular endothelial cells [4]. Klotho gene-deficient mice exhibit phenotypes similar to several human aging characteristics, such as reduced lifespan, sexual dysfunction, emphysema, deafness, skin atrophy, and system dysfunction of the kidneys, bone, marrow, blood, and brain [1].

2,3,5,4′-Tetrahydroxystilbene-2-O-*β*-D-glucoside (THSG), a resveratrol analog of glucoside, exhibits many biological and pharmacological activities [5]. Orally administered THSG is mainly distributed in the heart, kidneys, liver, and lungs of rats [6], and THSG levels in the plasma and tissues of rats range from 0.27 to 185.00 *μ*g/mL [7]. Regarding cytotoxicity, the hepatotoxicity of THSG had not been determined in hepatic cells for a concentration of 400 *μ*mol/L [8] and in rat livers for 300 mg/kg for 60 days [9]. THSG can extend the lifespan of* Caenorhabditis elegans* [10]. Our previous studies have determined that THSG suppresses aortic *β*-galactosidase (senescence-associated *β*-galactosidase [SA-*β*-gal]) activity, histone H2A histone family member X (*γ*H2AX) phosphorylation, and p53 acetylation in spontaneously hypertensive rats (SHRs). THSG promotes sirtuin 1 (SIRT1) activity [11, 12] and stimulates endothelial nitric oxide synthase (eNOS) gene luciferase reporter activity and eNOS protein expression. These findings demonstrate that THSG can prevent vascular senescence and improve blood flow through the SirT1–eNOS axis, at least in part, in vitro and in vivo [11]. In the present study, we tested whether THSG regulates the expression of klotho in vitro and in vivo and its related function.

## 2. Materials and Methods

### 2.1. Cell Culture

Human Embryonic Kidney 293 (HEK-293) cells were cultured in high-glucose Dulbecco's modified Eagle's medium (DMEM), or NRK-52E renal tubular epithelial cells were cultured in DMEM medium, containing 10% fetal bovine serum. Cells were grown in a 10 cm dish in 37°C incubator (5% CO_2_). The medium was changed every other day, and cells were grown to 80%–90% confluence and digested with 0.25% trypsin and subcultured.

### 2.2. Animals and THSG Administration

Male SHRs and Wistar-Kyoto rats (WKYs) aged 12 weeks were purchased from Beijing Vital River Laboratory Animal Technology Co., Ltd. (Beijing, China). The animals were handled in compliance with the Guide for the Care and Use of Laboratory Animals, and the animal experiments in this study were approved by the Animal Ethics Committee of Shanghai University of Traditional Chinese Medicine. The body weight and rat tail blood pressure were measured before group allocation to ensure homogeneity. Rats were housed in cages (3 per cage) at constant humidity (65%  ± 5%) and temperature (24°C ± 1°C) in a 12-hour light-dark cycle, and rats were provided ad libitum access to tap water and food throughout the experiments. SHRs were adapted to the environment for 1 week and then randomly divided into the following 3 groups (8 per group): 12-week-old control group, 30-week-old control group, and THSG treatment group. Rats in the 12-week-old control group were reared for 12 weeks and then dissected. THSG at 50 mg/kg per day was orally administered to rats in the THSG treatment group for 18 weeks. Rats in the 30-week-old control SHR group received equivalent amounts of adjuvant sodium carboxymethyl cellulose. On the last day of treatment, rats were anesthetized by an intraperitoneal injection of pentobarbital at a dose of 40 mg/kg of body weight and dissected, and their organs were isolated. After weighing, the organs were frozen in liquid nitrogen and stored at −80°C.

### 2.3. Detection of Luciferase Reporter System

HEK-293 cells were seeded in 24-well plates at a density of 2 × 10^5^ cells/well. The cells were cultured overnight and grown to 70%–90% confluence for transfection with plasmids. In each well, 0.8 *μ*g of a pGL3 reporter plasmid containing a klotho promoter (from −1000 to +299) and the firefly luciferase gene and 0.016 *μ*g of a reporter pRL-SV40 plasmid containing the* Renilla* luciferase gene as a normalization control were transfected and diluted with the FuGENE HD transfection reagent in the Opti-MEM transfection medium. The medium was replaced with normal DMEM 6 h after transfection. After incubation with THSG (purity > 98%, Shanghai Ding Jie Biological Technology Co., Ltd., Shanghai, China) for 16 h, the activity of the luciferase reporter gene was assayed using the dual-luciferase reporter 1000 assay system and detected using a Varioskan Flash microplate spectrophotometer (Thermo Scientific, USA).

### 2.4. RNA Isolation and Quantitative Polymerase Chain Reaction

Total RNA was extracted from rat tissues or NRK-52E cells by using Invitrogen's TRIzol reagent (Life Technologies, Grand Island, USA) according to the manufacturer's instructions. First-strand cDNA was synthesized using the High Capacity cDNA Reverse Transcription Kit (Life Technologies). The obtained cDNA was mixed with the Maxima SYBR Green qPCR Master Mix (Thermo Scientific, Waltham, MA, USA) and gene-specific primers (Shanghai Generay Biotech, Shanghai, China). The primer sequences are listed in Table 1. Quantitative polymerase chain reaction (qPCR) was performed using the Applied Biosystems 7500 Fast Real-Time PCR System (Thermo Scientific) according to the manufacturer's instructions. The amplification conditions consisted of an initial denaturation step at 95°C for 15 min, followed by 40 cycles of denaturation at 95°C for 15 s, annealing at 58°C for 30 s, and elongation at 72°C for 30 s. The dissociation curves were analyzed to ensure the amplification of a single PCR product. Three independent assays were performed for each primer. The amount of cDNA was calculated for each sample from the standard curve. The relative expression was determined after the normalization of gene expression of glyceraldehyde-3-phosphate dehydrogenase (GAPDH).

### 2.5. Western Blotting

NRK-52E cells were lysed in ice-cold Radio Immunoprecipitation Assay (RIPA) buffer (50 mmol/L Tris/HCl, pH 8.0; 150 mmol/L NaCl; 2 mmol/L sodium orthovanadate; 1% Nonidet-P40; 1% sodium deoxycholate; 0.1% sodium dodecyl sulfate [SDS], 0.1 mmol/L DTT; 0.05 mmol/L PMSF; 0.002 mg/mL aprotimin; and 0.002 mg/mL leupeptin). Lysates were centrifuged at 12000 rpm/min for 10 min at 4°C. Aliquots of the cell lysate (50 or 100 *μ*g of each sample) were resolved on SDS-polyacrylamide gel electrophoresis and transferred to nitrocellulose membranes. The membranes were blocked in 5% skim milk overnight at 4°C. The membranes were incubated with primary antibodies for 2 h and then with a horseradish peroxidase- (HRP-) conjugated secondary antibody at room temperature for 1 h. The bands were visualized using an ECL Immobilon Western Chemiluminescent HRP substrate (Millipore, Billerica, MA, USA). Quantitative analysis of band density was performed using Quantity One software from Bio-Rad (Hercules, CA, USA). Western blotting was performed in triplicate.

### 2.6. Immunohistochemical Staining

Formalin-fixed, paraffin-embedded tissue blocks were cut into 6 *μ*m sections, followed by staining. Immunohistochemical staining was performed using mouse anti-8-hydroxydeoxyguanosine (anti-8-OHDG; ab62623, Abcam, Cambridge, UK) and rabbit anti-klotho primary antibodies (ab154163, Abcam), with goat anti-mouse (sc-2005, Santa Cruz Biotechnology, California, CA, USA) and goat anti-rabbit (A0208, Beyotime, China) secondary antibodies, and the LSAB 2 System-HRP was used as the Rat Specimens Kit (Dako, Hamburg, Germany). The intensity of immunohistochemical staining was analyzed using an ImageJ analysis system (NIH, Maryland, MD, USA).

### 2.7. Cell Senescence Model and Senescence Staining

NRK-52E cells were grown to 80%–90% confluence and digested with 0.25% trypsin. Cells were seeded in 24-well plates at a density of 5 × 10^5^ cells/well. After adherence, cells were stimulated with 100 nmol/L angiotensin II (Ang II) for 4 days, and the medium including Ang II was changed every 24 h. Subsequently, the cells were stained to assess SA-*β*-gal activity by using the Cellular Senescence Assay Kit (Cell Biolabs, Inc., San Diego, CA, USA). NRK-52E cells were fixed in 1% formaldehyde containing 0.2% glutaraldehyde in phosphate-buffered saline (PBS) for 5 min at room temperature, rinsed with PBS, and incubated with fresh *β*-galactosidase staining solution (1 mg/mL 5-bromo-4-chloro-3-indolyl *β*-D galactoside, 40 mmol/L citric acid/sodium phosphate [pH 6.0], 5 mmol/L of potassium ferrocyanide, and 150 mmol/L of MgCl_2_) at 37°C for 4 h or overnight. After staining, the working fluid was discarded, and samples were washed 2 times with PBS. Aging cells were imaged and counted under an optical microscope.

### 2.8. Determination of Serum Malondialdehyde

Serum malondialdehyde (MDA) levels were measured using a kit from the Nanjing Institute of Biological Engineering (Nanjing, China) in accordance with the procedure specified by the manufacturer. After the appropriate sample and the reagent were added and mixed thoroughly, a small hole was pricked in the lids of centrifuge tubes by using a needle, and samples were heated to 95°C in a water bath for 80 min. Samples were removed from the water bath, cooled under running water, and centrifuged at 4000 rpm/min for 10 min. A 200 *μ*L aliquot of the supernatant was seeded into a 96-well plate, and the optical density (OD) of each well was detected at a wavelength of 532 nm by using the SpectraMax 190 Absorbance Microplate Reader (Molecular Devices Corporation, Sunnyvale, CA, USA). Serum MDA levels (nmoL/mL) were calculated as follows: (sample OD value − blank OD value)/(standard OD value − blank OD value) × standard concentration (10 nmoL/mL) × sample dilution before testing.

### 2.9. Glutathione Peroxidase Activity Assay

Serum glutathione peroxidase (GSH-Px) activity was measured using a kit from the Nanjing Institute of Biological Engineering (Nanjing, China) in accordance with the procedure specified by the manufacturer. The appropriate sample and the reagent were added and mixed thoroughly to facilitate the enzyme-catalyzed reaction. Samples were centrifuged at 4000 rpm/min for 10 min. A 0.1 mL aliquot of the supernatant was added to another test tube. For the color reaction, the chromogenic agent was added and mixed thoroughly. After standing at room temperature for 15 min, the OD of each well was detected at a wavelength of 520 nm by using a Microplate Reader. Serum glutathione peroxidase (GSH-Px) activity was calculated as follows: (control OD value − sample OD value)/(standard OD value − blank OD value) × standard concentration (20 *μ*moL/mL) × sample dilution (6 times) × sample dilution before testing.

### 2.10. Alkaline Phosphatase Activity Assay

Serum alkaline phosphatase activity was measured using a kit from the Nanjing Institute of Biological Engineering (Nanjing, China) in accordance with the procedure specified by the manufacturer. The appropriate sample and the reagent were added, mixed thoroughly, and incubated in a water bath at 37°C for 15 min. Finally, the chromogenic agent was added to each well, gently shaken, and mixed thoroughly, and the OD of each well was determined at a wavelength of 520 nm. Serum alkaline phosphatase activity (Guinness units/100 mL) was calculated as follows: (sample OD value − blank OD)/(standard OD value − blank OD value) × standard concentration (0.1 mg/mL) × 100 mL × sample dilution before testing.

### 2.11. Determination of Serum Calcium and Phosphorus

The rat serum was mixed thoroughly with 0.4 mL of the protein precipitating agent, centrifuged at 3500 rpm/min for 10 min, and placed in an ice bath until use. Serum calcium and phosphorus levels were measured using a kit from the Nanjing Institute of Biological Engineering (Nanjing, China) in accordance with the procedure specified by the manufacturer. Serum calcium and phosphorus levels were measured through the methyl thymol blue method and the molybdenum blue method, respectively. The OD of each well was determined at a wavelength of 610 nm (calcium) or 660 nm (phosphorus) by using a Microplate Reader. Serum calcium and phosphorus levels were calculated as follows: serum calcium or phosphorus level (mmol/L) = (sample OD value − blank OD value)/(standard OD value − blank OD value) × standard concentration (1 mmol/L) × sample dilution before testing.

### 2.12. Determination of Bone Mineral Density, Mineral Salt, and Strength

After removing the muscles and ligaments attached to the femur and keeping the femur clean and dry, the bone mineral density and mineral salt content of the left femur of the rats were determined using Lunar DPX-L Dual Energy X-ray Absorptiometry Equipment (General Electric, New York, USA). Alternatively, the femoral bearing strength or the maximum transverse bearing strength of bone fracture or the fractured right femur of rats was measured using a bone strength measuring device (Jinan Yi Yan Technology Development Co., Ltd., Jinan City, China).

### 2.13. Statistical Analyses

The results are presented as means ± SEM. For parametric data, comparisons among treatment groups were performed using analysis of variance, followed by post hoc tests. All analyses were performed using SPSS (SPSS, Inc., Northampton, MA, USA). *P* < 0.05 was considered statistically significant.

## 3. Results

### 3.1. THSG Regulated Klotho Expression and Improved Angiotensin II-Induced Cell Senescence In Vitro

**Figure 1:**
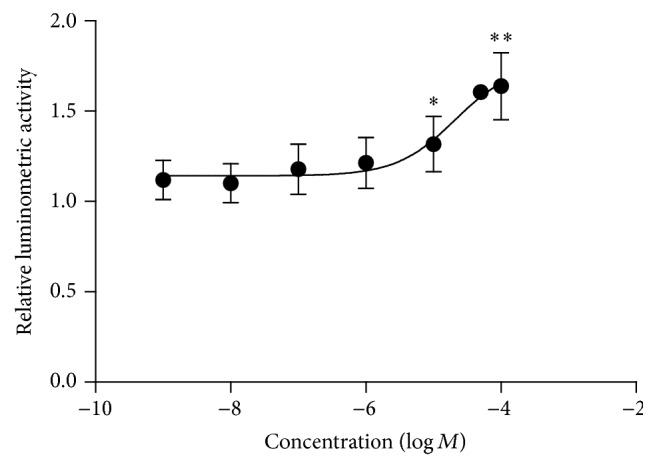
Effect of THSG on the luciferase reporter activity of the klotho gene. HEK-293 cells that were transiently transfected with luciferase reporter plasmids driven by a human klotho promoter (from −1000 to +299) and reporter pRL-SV40 plasmid containing the* Renilla* luciferase gene as normalization were stimulated with the indicated doses of THSG for 16 h. Cells were lysed and analyzed for luciferase activity (*n* = 3). Data are expressed as mean ± SD. ^*∗*^ *P* < 0.05 versus control empty plasmid, and ^*∗∗*^ *P* < 0.01 versus control empty plasmid.

To observe the effects of THSG on klotho gene expression, we constructed a pGL3 basic plasmid containing a human klotho promoter (from −1000 to +299) and the firefly luciferase gene, which was transfected into HEK-293 cells. The cells were treated with different concentrations of THSG (10^−6^–10^−4^ mol/L) for 16 h. The luciferase reporter activity of the klotho gene was determined to be increased in a dose-dependent manner (Figure 1), with EC_50_ of 21 *μ*mol/L.

**Figure 2:**
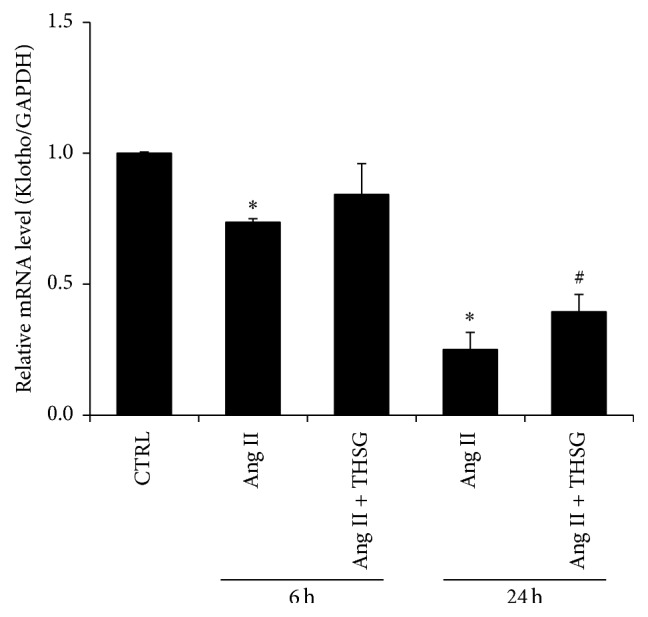
THSG reversed the decrease in klotho mRNA expression mediated by Ang II in NRK-52E rat renal tubular epithelial cells in vitro. Cells were stimulated with or without Ang II (100 nmol/L) for 6 or 24 h and were treated with or without THSG for 6 or 24 h. Klotho mRNA expression was measured using the Maxima SYBR Green qPCR Master Mix. Data are normalized to GAPDH mRNA expression levels (*n* = 3 per group). Data are expressed as mean ± SD. ^*∗*^ *P* < 0.05 versus control group; ^#^ *P* < 0.05 versus Ang II-stimulated group.

**Figure 3:**
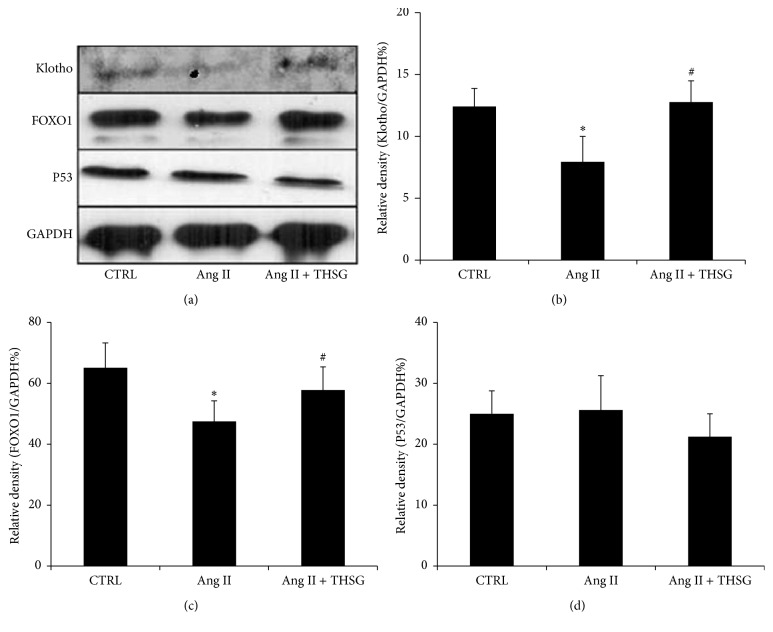
THSG reversed the decrease in klotho and FOXO1 protein expression mediated by Ang II in NRK-52E rat renal tubular epithelial cells in vitro. Cells were stimulated with or without Ang II (100 nmol/L) for 24 h and treated with or without THSG for 24 h. The expression of proteins was detected using Western blotting, and relative densities were normalized to GAPDH expression levels (each group *n* = 3). Data are expressed as mean ± SD. ^*∗*^ *P* < 0.05 versus control group; ^#^ *P* < 0.05 versus Ang II-stimulated group.

Renal tubular epithelial cells are one of the main cells with klotho expression. We studied the effect of THSG on klotho expression in NRK-52E rat renal tubular epithelial cells. Klotho mRNA expression was suppressed after cells were stimulated with 100 nmol/L Ang II for 6 or 24 h (Figure 2). However, treatment of cells simultaneously with THSG reversed Ang II-induced inhibition of klotho mRNA expression (Figure 2). Furthermore, after cells were stimulated with Ang II for 24 h, klotho protein expression was reduced, and THSG reversed this trend (Figure 3). In addition, THSG reversed the Ang II-induced inhibition of the protein expression of forkhead box O1 (FOXO1), another aging-related gene. However, p53 protein expression remained unaffected (Figure 3).

**Figure 4:**
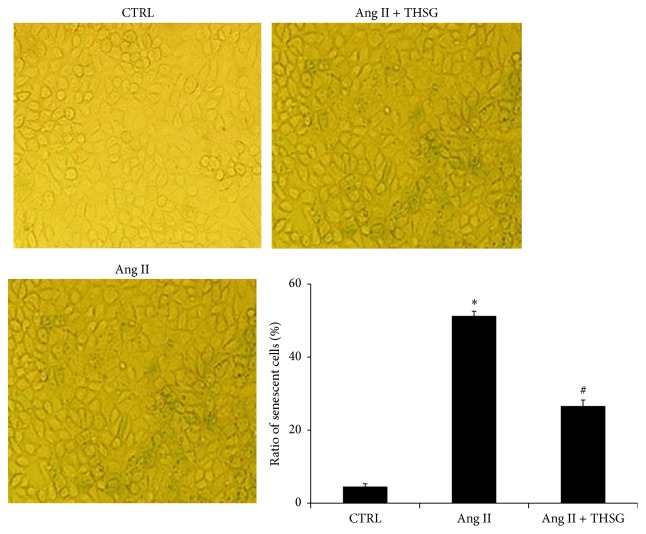
THSG inhibited the Ang II-induced senescence of NRK-52E rat renal tubular epithelial cells. Cells were stimulated with or without Ang II (100 nmol/L) for 4 days and treated with or without THSG for 4 days. Subsequently, cells were stained using a *β*-galactosidase senescence assay kit. More than 200 cells were counted in each sample, and the proportion of senescent cells was calculated (*n* = 3 per group). Data are expressed as mean ± SD. ^*∗*^ *P* < 0.05 versus control group; ^#^ *P* < 0.05 versus Ang II-stimulated group.

Furthermore, long-term stimulation of cultured cells with Ang II (4 days) resulted in the senescence of NRK-52E renal tubular epithelial cells, with nearly 50% of the cell population comprising aging cells (Figure 4). However, THSG treatment significantly reduced the proportion of aging cells, and more than 20% of the cell population comprised aging cells (Figure 4).

### 3.2. THSG Regulated Klotho Expression and Enhanced Antioxidant Capacity and Bone Strength In Vivo

No significant difference was observed in the blood pressure, the body weight growth trend, and the ratios of organ weight/body weight between 30-week-old SHRs and THSG-treated rats (data not shown).

**Figure 5:**
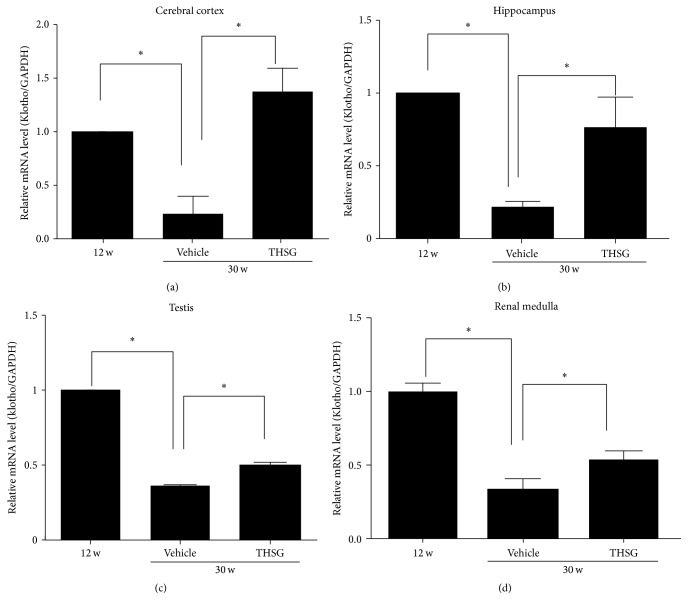
Effect of THSG on klotho expression in SHRs. Total RNA was extracted from the rat cerebral cortex, hippocampus, testis, and renal medulla by using Invitrogen's TRIzol reagent. Klotho mRNA expression was measured using the Maxima SYBR Green qPCR Master Mix. Data were normalized to GAPDH mRNA expression levels (each group *n* = 8). Data are expressed as mean ± SD; ^*∗*^ *P* < 0.05.

**Figure 6:**
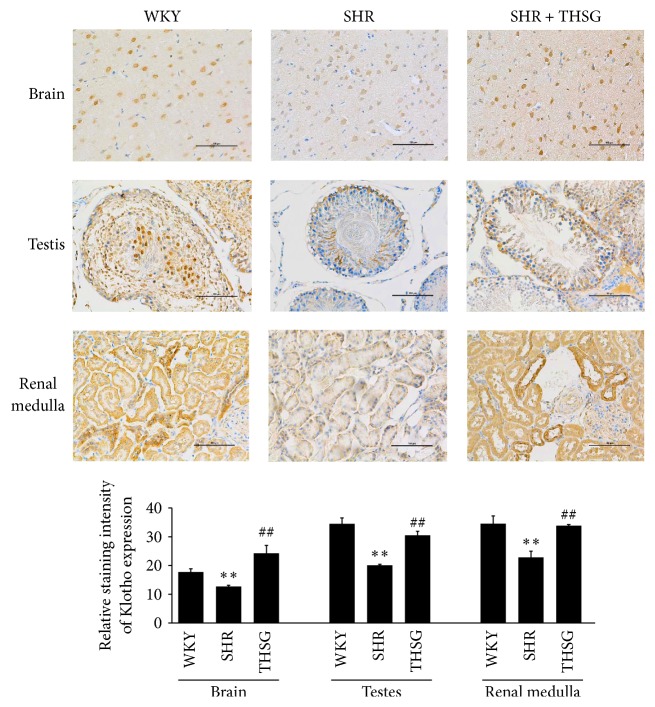
Klotho expression in the brain, testis, and renal medulla. Immunohistochemical staining for klotho was performed on tissues from 30-week-old WKY rats, 30-week-old SHRs, and SHRs that were treated with THSG. The values in the bar graphs represent the means ± SD; *n* = 3. ^*∗∗*^ *P* < 0.01 versus the WKY group; ^##^ *P* < 0.01 versus the SHR group.

Klotho mRNA expression was detected in the heart, liver, spleen, renal medulla, renal cortex, adrenal gland, cerebral cortex, hippocampus, bladder, testis, and prostate of 30-week-old SHRs, and klotho expression was significantly reduced in the cerebral cortex, hippocampus, testis, and renal medulla compared with 12-week-old SHRs (each *n* = 8; *P* < 0.01; Figure 5). THSG administration for 18 weeks increased klotho mRNA expression in the cerebral cortex, hippocampus, testis, and renal medulla of 30-week-old SHRs (each *n* = 8; *P* < 0.01; Figure 5). Similar to the aforementioned results determined using qPCR, tissue immunostaining revealed that klotho expression was lower in 30-week-old SHRs than in 30-week-old WKY rats. Furthermore, THSG administration restored klotho expression in the brain, testes, and renal medulla (Figure 6).

**Figure 7:**
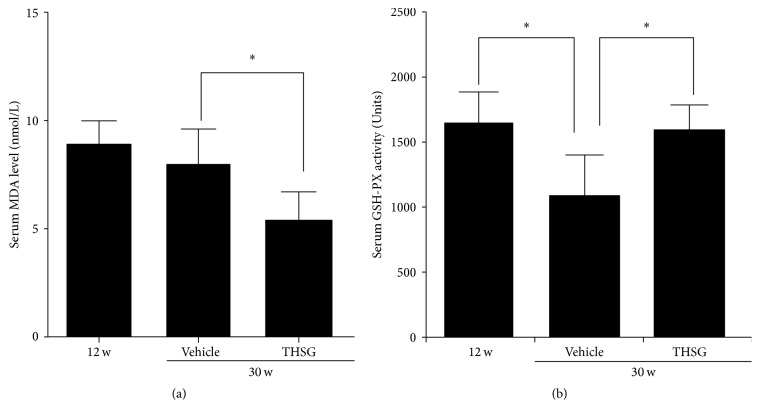
Antioxidant capacity of THSG in SHR sera. Sera were obtained from 12-week-old and 30-week-old SHRs and 30-week-old SHRs that were treated with THSG (*n* = 8 per group). (a) Lipid-peroxidation MDA and (b) GSH-Px activity were determined in accordance with the procedures of the kit manufacturer. Data are expressed as mean ± SD; ^*∗*^ *P* < 0.01.

**Figure 8:**
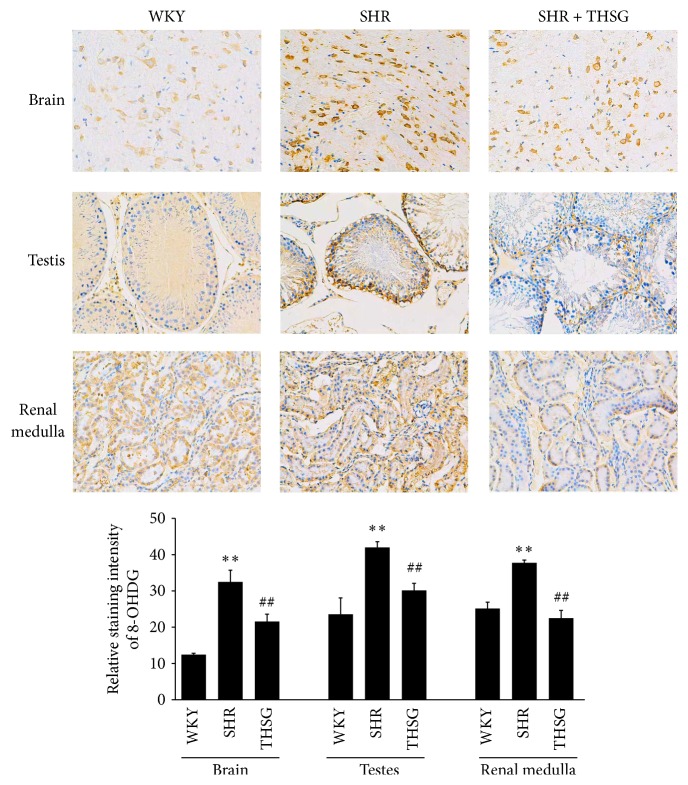
8-OHDG levels in the brain, testis, and renal medulla. Immunohistochemical staining for 8-OHDG was performed on tissues from 30-week-old WKY rats, 30-week-old SHRs, and SHRs that were treated with THSG. The values in the bar graphs represent the means ± SD; *n* = 3. ^*∗∗*^ *P* < 0.01 versus the WKY group; ^##^ *P* < 0.01 versus the SHR group.

THSG increased the blood antioxidant capacity, reduced MDA levels, and increased serum GSH-Px activity (each *n* = 8, compared with 30-week-old SHRs; *P* < 0.01; Figure 7). The level of 8-OHDG, a marker of oxidized nucleosides in DNA, was higher in the tissue sections of the brain, testes, and renal medulla of 30-week-old SHRs than in 30-week-old WKY rats. THSG reduced the upward trend of the 8-OHDG level (Figure 8). THSG also reversed the downward trend in serum calcium in SHRs (*n* = 8, compared with 30-week-old SHRs; *P* < 0.05; Table 2). Although no significant change was observed in femoral mineral density and bone mineral content in each group of SHRs, femoral bearing strength increased in the THSG treatment group (*n* = 8, compared with 30-week-old SHRs; *P* < 0.05; Table 2). In addition, although there were no statistically significant differences, THSG was found to have a tendency to increase creatinine clearance and decrease urinary protein excretion (data not shown).

## 4. Discussion

### 4.1. Hypertension and Angiotensin II Inhibited Klotho Expression and the Effect of THSG

This study demonstrated that Ang II stimulation and hypertension inhibited klotho expression (Figures 2, 3, and 5). One study reported that Ang II plays a role in inhibiting klotho expression [13], which is consistent with our findings. Ang II is a crucial inducer of inflammation and oxidative stress, and it can activate NF-*κ*B transcription activity [14–16] and mitochondrial superoxide [17–19] in different cell types. Moreover, NF-*κ*B activation and reactive oxidative stress downregulate klotho expression [20–22]. Long-term hypertension is a cardiovascular disease that causes stroke, cardiac hypertrophy, renal failure, and organ fibrosis complications. Various animal models of human hypertension, SHRs, deoxycorticosterone acetate-salt hypertensive rats, and 5/6 nephrectomized rats indicate that sustained circulatory stress inhibits klotho expression [23]. Similarly, hypertensive rats also exhibit low-grade inflammation and harbor high-degree oxidative stress products and present enhanced superoxide production [24–26] and NF-*κ*B activation [27, 28]. In the present study, THSG could reverse the inhibitory effect of Ang II and hypertension on klotho expression (Figures 2, 3, and 5). Additionally, THSG-induced klotho expression appeared to be direct and dose-dependent in the luciferase reporter test (Figure 1). Although the mechanism by which THSG directly regulates the expression of klotho remains unclear, many studies have indicated that THSG can inhibit oxidative stress and NF-*κ*B activation [29–32].

### 4.2. Impact of Improved Klotho Expression in the Brain, Kidneys, and Testis

Analysis of klotho mRNA expression in many tissues revealed that mRNA expression was decreased in the brain, testis, and renal medulla of aged SHRs, and the decrease was reversed by THSG (Figures 5 and 6). This finding suggests tissue specificity when THSG regulates the expression of klotho. Augmenting klotho or its effects may enhance cognition and counteract cognitive deficits at different life stages [33, 34], promote the vitality of spermatogenesis [35], and ameliorate aging-related, Ang II-induced, or glomerulonephritis renal injury [36–38]. Previous studies have shown that THSG can improve learning and memory and promote hippocampal synaptic plasticity in aged rats [39, 40], which may contribute to regulating klotho expression by THSG.

### 4.3. THSG Antioxidant Effect and Calcium Homeostasis

Our results indicated that THSG could reduce Ang II-induced NRK-52E renal tubular epithelial cell senescence (Figure 4). Our previous study demonstrated that THSG inhibited the senescence of human and rat vascular endothelial cells and the thoracic aortas in SHRs [11]. Our results also showed that THSG enhanced the antioxidant capacity in vivo, reduced serum MDA and tissue 8-OHDG levels, and increased serum GSH-Px activity (Figures 7 and 8) in aged SHRs. Similar to our results, previous studies have demonstrated that THSG can inhibit oxidative stress in other rat and mouse models [41–43]. In addition, the klotho protein is involved in regulating the antioxidative defense [34]. THSG increased blood calcium levels and the femoral bearing strength (Table 2), indicating that THSG regulates calcium homeostasis by increasing the klotho protein level. Previous studies have reported that klotho can regulate calcium homeostasis [44, 45] and bone metabolism [46, 47]. One cell-level study reported that the protective effect of THSG in osteoblastic MC3T3-E1 cells was mediated by inhibiting the release of bone-resorbing mediators and oxidative damage to cells [32], reflecting another effect of THSG.

## 5. Conclusion

This study demonstrated that THSG regulates klotho expression at the molecular, cellular, and in vivo levels by using the luciferase reporter activity assay, quantitative real-time PCR, and Western blotting. This study also determined that THSG delays cell aging, improves antioxidant capacity, and protects the bone. Because THSG reversed the low expression of klotho in the brain, kidneys, and testis of SHRs, THSG may enhance cognition at different life stages, promote the vitality of spermatogenesis, and ameliorate the renal injury and the antioxidant and bone metabolism that are mediated by klotho.

## Figures and Tables

**Table 1:** Primer sequences of target genes and annealing temperature.

Gene	Primer sequences	Annealing temperature
Klotho (138 bp)	Sense: 5′-ACTTTCTTCTGCCCTATTTCACG-3′	58°C
	Antisense: 5′-CCAGGTAATCGTTGTATTTTATCGG-3′	
GAPDH (192 bp)	Sense: 5′-GATCCCGCTAACATCAAATG-3′	58°C
	Antisense: 5′-GAGGGAGTTGTCATATTTCTC-3′	

**Table 2:** Effect of *Polygonum* stilbene glucoside on serum calcium and phosphorus levels and alkaline phosphatase activity and bone strength (*x* ± *S*).

Groups	Serum calcium	Serum phosphorus	Alkaline phosphatase	Femur BMD	Bone mineral content	Femur bearing strength
	(mmol/L)	(mmol/L)	(IU/L)	(g/cm^2^)	(g/cm^2^)	(kg)
SHR (12 w)	2.18 ± 0.14	0.83 ± 0.27	74.1 ± 12.4	0.167 ± 0.019	0.475 ± 0.044	10.60 ± 2.03
SHR (30 w)	1.90 ± 0.17^*∗*^	0.51 ± 0.24^*∗*^	73.9 ± 29.9	0.219 ± 0.013^*∗*^	0.685 ± 0.098^*∗*^	12.79 ± 1.21^*∗*^
SHR (30 w) + THSG	2.11 ± 0.16^#^	0.46 ± 0.12	65.2 ± 17.6	0.228 ± 0.014	0.640 ± 0.075	14.19 ± 1.38^#^

^*∗*^Compared with 12-week-old SHRs, *P* < 0.05. ^#^Compared with 30-week-old SHRs, *P* < 0.05

## Abbreviations

Ang II: Angiotensin II
DMEM: Dulbecco's modified Eagle's medium
eNOS: Endothelial nitric oxide synthase
FOXO1: Forkhead box O1
GAPDH: Glyceraldehyde-3-phosphate dehydrogenase
GSH-Px: Glutathione peroxidase
*γ*H2AX: H2A histone family member X
HEK-293 cell: Human embryonic kidney 293 cell line
HRP: Horseradish peroxidase
MDA: Malondialdehyde
NRK-52E: Rat renal tubular epithelial cell line
8-OHDG: 8-Hydroxydeoxyguanosine
p53: Tumor protein p53
qPCR: Real-time quantitative PCR detecting
SA-*β*-gal: *β*-Galactosidase
SHR: Spontaneously hypertensive rat
WKY: Wistar-Kyoto rat
SIRT1: Sirtuin 1
THSG: 2,3,5,4′-Tetrahydroxystilbene-2-O-*β*-D-glucoside.
